# Bioequivalence study of cyclosporine in renal transplant recipients

**DOI:** 10.4103/0970-1591.36730

**Published:** 2007

**Authors:** Gagan Gautam

**Affiliations:** Department of Urology and Renal transplant, Fortis Flt. Lt. Rajan Dhall Hospital, Sector B, Pocket 1, Aruna Asaf Ali Marg, Vasant Kunj, New Delhi - 110 070, India. E-mail: gagangg@gmail.com

Dear Sir,

I read with interest the article by Jha *et al*.[1]

In the discussion, the authors have graciously accepted a few limitations of their study. They state: “Some limitations of this study include the small sample size which increases the probability of making a Type II error….”

This statement is statistically erroneous since the statistical treatment of bioequivalence studies is different from the studies which simply compare the efficacy of two different therapeutic options.[2]

Birkett defined bioequivalence by stating that, “two pharmaceutical products are bioequivalent if they are pharmaceutically equivalent and their bioavailabilities (rate and extent of availability) after administration in the same molar dose are similar to such a degree that their effects, with respect to both efficacy and safety, can be expected to be essentially the same”.[3]

Bioequivalence testing is conducted by obtaining serum plasma samples at regular intervals and assaying for the drug concentration. This procedure has been performed in the aforementioned study by assaying the Cyclosporine (C_2_) levels on Days 1, 4, 7, 15, 30 and 90 after switchover from Neoral or Bioral to Imusporin. This was the primary efficacy parameter of the study. Occasionally, blood concentration levels are neither feasible nor possible to compare the two products (e.g. inhaled corticosteroids), then pharmacodynamic endpoints rather than pharmacokinetic endpoints are used for comparison. These factors have been additionally included as secondary end points (incidence of graft rejection and changes in serum creatinine) in the study.[1]

Statistically speaking, since the objective is to prove that two formulations are bioequivalent, the null hypothesis in this case is the opposite i.e. “The two different formulations are not bioequivalent.” Type II error (error of omission) is the lesser of the two evils and can only occur when there is a failure to reject the null hypothesis. In this example, a Type II error will occur when we state that the two preparations are not bioequivalent when in fact they are. If we have concluded that these two preparations (Neoral/Bioral and Imusporin) are bioequivalent, as is the case in this study, the error that we may commit is indeed the Type I error (error of commission) which is more dangerous in the statistical as well as the clinical scenario. We would be committing a Type I error if we state that the preparations are bioequivalent when in fact they are not.

Bioequivalence studies need exceptional statistical treatment and the results need to be interpreted with care. There are special tests, like the two one sided testing procedure, which are specifically suited to such study designs. Statistical conclusions on the basis of conventional tests of significance (like the paired t-test) may not be completely appropriate and relevant in such circumstances.
